# The overlooked burden: associations between high-noise environments and scrub nurse wellbeing and performance

**DOI:** 10.3389/fpubh.2026.1812411

**Published:** 2026-05-28

**Authors:** Yue Ma, Tingting Ni, Long Pang, Yi Luo

**Affiliations:** 1 Department of Ultrasound, West China Hospital, Sichuan University, Chengdu, China; 2 West China School of Nursing, Sichuan University, Chengdu, China; 3 Operating Room of Anesthesia Surgery Center, West China Hospital, Sichuan University, Chengdu, China; 4 Department of Orthopedics, Orthopedic Research Institute, West China Hospital, Sichuan University, Chengdu, China

**Keywords:** operating room noise, physiological impact, psychological health, scrub nurses, surgical performance

## Abstract

**Objective:**

Operating room noise is a significant occupational hazard affecting healthcare professionals, but the specific consequences for scrub nurses have been largely overlooked. This study aimed to investigate the effects of operating room noise on scrub nurses across three key domains: psychological status, physiological and physical symptoms, and perceived surgical performance.

**Methods:**

A cross-sectional comparative study was conducted among 94 scrub nurses working in high-noise (orthopedic) and low-noise (ophthalmic) operating rooms. Participants completed validated psychological scales, underwent standardized physiological measurements, and reported physical symptoms and perceived noise impact on surgical performance. Between-group comparisons were performed using IBM SPSS Statistics 26.0 (*P* < 0.05).

**Results:**

Psychologically, the high-noise group reported significantly higher levels of anxiety, sleep disturbances, specific burnout dimensions (exhaustion and cynicism), and perceived stress compared to the low-noise group (*P* < 0.05). Physiologically, nurses in the high-noise environment exhibited elevated resting heart rates (RHR) and systolic blood pressure (SBP). Furthermore, they reported a significantly higher prevalence of noise-related physical symptoms, including tinnitus and palpitations, rather than clinically diagnosed conditions. Regarding performance, the high-noise group perceived a substantially greater negative impact of noise on their task efficiency, surgical concentration, and intraoperative communication.

**Conclusion:**

The findings suggest that prolonged exposure to high-noise environments is associated with elevated psychological distress, physiological stress responses, and frequent somatic complaints among scrub nurses. Additionally, high noise levels are perceived to negatively interfere with surgical performance. Longitudinal studies and targeted noise-reduction interventions are recommended to safeguard occupational health and patient safety.

## 1 Introduction

Modern operating rooms are getting increasingly noisy, with sounds from equipment, monitors, heating ventilation and air conditioning (HVAC) systems, music, and team communication contributing to noise pollution (1–5). The World Health Organization (WHO) and Environmental Protection Agency (EPA) recommend noise limits of 35 A-weighted decibels (dBA) and 45 dBA for operating rooms, respectively (6). However, studies have showed that operating room noise levels often exceed these safety standards, with average levels ranging from 53 dBA to 93 dBA, particularly during orthopedic surgeries (1, 7–9).

Given these high noise levels, current literature has reported the negative impact of operating room noise on both healthcare professionals and patients. Exposure to high noise levels can negatively affect surgical performance (10, 11) and cognitive functions such as attention, memory, and decision-making for surgeons (10, 12). In addition, noises have been associated with exhaustion (13), stress (13), impaired communication (14, 15), higher error rates (16, 17) and the risk of noise-induced hearing loss (NIHL) (10, 18–20). Patients are also affected by the noises, which can lead to increased physiological stress (21), sleep disturbances (21), delayed recovery (22), and a higher risk of complications (22–24). However, existing studies have primarily focused on the impact of operating room noise on surgeons, anesthesiologists, and patients, leaving the experiences of scrub nurses largely unexamined. This represents a critical gap considering scrub nurses play important roles in ensuring intraoperative safety and efficiency.

This study aims to investigate the effects of operating room noise on scrub nurses by examining three key domains: psychological status, physiological and physical symptoms, and perceived surgical performance. We hypothesize that scrub nurses in high-noise environments will exhibit higher levels of psychological distress and physiological stress, report more noise-related physical complaints, and perceive a greater negative impact on their intraoperative performances compared to those in low-noise environments.

## 2 Methods

### 2.1 Ethical considerations

This study was approved by the Institutional Review Board of West China Hospital, Sichuan University (Approval No.: 2024-1119), and was conducted in accordance with the Declaration of Helsinki. Informed consent was obtained from all participants, and their confidentiality and anonymity were strictly maintained throughout the study.

### 2.2 Study design

This study adopted a cross-sectional, comparative design conducted within the surgical center of our hospital, focusing on scrub nurses working in orthopedic and ophthalmic operating rooms. The orthopedic rooms were defined as the high-noise environment, involving noise-intensive procedures such as joint arthroplasty, spinal surgery, and trauma fixation that frequently require powered instruments. The ophthalmic rooms served as the low-noise environment, primarily involving microscopic procedures such as cataract and retinal surgeries with minimal acoustic disturbance. This categorization was based on previous literature (1, 7–9) and further validated by on-site acoustic measurements.

Sound levels were measured during 10 representative orthopedic and 10 ophthalmic procedures at peak intraoperative activity, using a calibrated Type 2 integrating sound level meter (AWA5688, Hangzhou Aihua Instruments Co., Ltd., China) positioned approximately 1.5 m above the floor near the scrub nurse's typical working position. The orthopedic rooms exhibited substantially higher acoustic levels [equivalent continuous A-weighted sound pressure level (LAeq) = 68.2 ± 5.4 dBA; peak sound pressure level (Lpeak) = 108.5 dBA], with peaks frequently exceeding 90 dBA during the use of oscillating saws, drills, and reamers, and approximately 42% of operative time exceeding 70 dBA. In contrast, the ophthalmic rooms maintained low noise levels (LAeq = 51.5 ± 3.8 dBA; Lpeak = 72.4 dBA), with peaks rarely exceeding 70 dBA (< 2% of operative time). The between-group difference was statistically significant (*P* < 0.001), supporting the empirical validity of our noise classification and consistent with previous reports (1, 7–9).

### 2.3 Participant selection

Participants were selected according to the following inclusion criteria: (1) full-time scrub nurses; (2) employed at the surgical center for at least 24 months; (3) continuously working in the same type of operating room (high-noise or low-noise) for at least the preceding 12 months; and (4) working full-time day shifts to eliminate the confounding effects of circadian rhythm disruption.

To ensure that observed outcomes were primarily attributable to occupational noise exposure, strict exclusion criteria were applied: (1) congenital hearing impairments or a history of ear diseases; (2) history of significant non-occupational noise exposure (e.g., frequent attendance at loud music venues or regular use of headphones at high volumes); (3) pre-existing diagnosis of psychiatric disorders (e.g., depression, anxiety) or cardiovascular diseases (e.g., hypertension, arrhythmia); (4) current use of ototoxic drugs or medications affecting the psychological or cardiovascular systems (e.g., antidepressants, beta-blockers); (5) pregnancy or breastfeeding; and (6) refusal to participate.

### 2.4 Outcomes data collection

Data collection comprised three main components: self-reported psychological assessments, physiological measurements combined with self-reported noise-related physical symptoms, and subjective perception of noise impact. All data were collected anonymously using participant identification codes, and only group identifiers were retained for analysis. Although complete blinding of researchers to group allocation was not feasible due to the inherent visibility of operating room type and departmental affiliation, all psychological and symptom data were collected via standardized self-administered validated questionnaires, and physiological measurements were performed using a calibrated automated device following a uniform written protocol, thereby minimizing potential observer bias.

Self-reported psychological data were obtained using the following scales: Generalized Anxiety Disorder-7 (GAD-7) to assess anxiety (25, 26), Athens Insomnia Scale (AIS) for sleep disturbances (27), Maslach Burnout Inventory-General Survey (MBI-GS) to measure burnout (28), and the Perceived Stress Scale (PSS-10) to evaluate perceived stress (29). All these scales have demonstrated satisfactory psychometric properties in their Chinese versions, with well-established validity and reliability (30–33).

Physiological indicators include resting heart rate (RHR), systolic blood pressure (SBP), and diastolic blood pressure (DBP). These indicators were measured on a designated non-working day in the early morning between 7:00 and 9:00, with each participant scheduled at a consistent time of day to minimize circadian variability. Participants were instructed to (1) avoid caffeine, alcohol, and strenuous exercise for at least 24 h prior to measurement; (2) refrain from smoking and food intake for at least 30 min prior; and (3) empty their bladder before measurement. After at least 5 min of seated rest in a quiet, temperature-controlled room (22–24°C), three consecutive measurements were obtained at 1-min intervals using a calibrated electronic sphygmomanometer (Model HEM-7121, Omron Healthcare Co., Ltd., Kyoto, Japan), with an appropriately sized cuff applied to the non-dominant arm at heart level. The mean of the second and third readings was used in subsequent analyses. All measurements were performed by two trained research assistants following a standardized written protocol. Subjective physical symptoms were defined as self-reported health complaints experienced within the past 12 months. To minimize bias, symptoms were recorded as elf-reported health complaints experien≥2–3 times/week) and occurred during or within 2 h after surgical shifts. Evaluated symptoms included tinnitus, transient hearing difficulties, palpitations, gastrointestinal discomfort, headache, dizziness, and musculoskeletal discomfort.

Participants were also asked to rate their subjective perception of noise impact on task efficiency, surgical concentration, and intraoperative communication using a Visual Analog Scale (VAS) ranging from 0 to 10, where 0 indicated no adverse impact and 10 represented the maximum perceived adverse impact.

### 2.5 Sample size

The sample size was calculated using PASS 15.0 software (NCSS, LLC, Kaysville, UT, USA). We anticipated a mean difference of 5 points on the GAD-7 scale to be clinically significant. With an estimated standard deviation of 5.0 points, an alpha level of 0.05, a power of 0.80, and assuming equal group variances, the minimum required sample size was calculated to be 15 participants per group. To account for potential data invalidity or incomplete responses, the target sample size was increased, resulting in a final recruitment of 20 participants per group.

### 2.6 Data analysis

Data were analyzed using IBM SPSS Statistics for Windows, Version 26.0 (IBM Corp., Armonk, NY, USA). Continuous variables were expressed as mean ± standard deviation (SD) or median (interquartile range, IQR), depending on the normality of distribution assessed by the Shapiro-Wilk test. Comparisons between groups were performed using independent samples *t*-tests for normally distributed data and Mann-Whitney *U*-tests for non-normally distributed data. Categorical variables were presented as frequencies and percentages and compared using the chi-square (χ*2*) test or Fisher's exact test when expected cell counts were less than 5. To better reflect the magnitude and clinical relevance of between-group differences, effect sizes were calculated alongside *P*-values. For continuous outcomes, mean differences with 95% confidence intervals (CIs) and Cohen's *d* (with 95% CI) were reported, with *d* values of approximately 0.2, 0.5, and 0.8 interpreted as small, medium, and large effects, respectively. For categorical outcomes, odds ratios (ORs) with 95% CIs were calculated. A two-sided *P*-value of less than 0.05 was considered statistically significant.

## 3 Results

### 3.1 Baseline characteristics of participants

A total of 173 scrub nurses (91 from orthopedics and 82 from ophthalmology) were screened. After applying the inclusion and exclusion criteria, 94 nurses (46 from orthopedics and 48 from ophthalmology) were eligible. The two groups were comparable in terms of age, BMI, gender, work experience, weekly work hours, education level, and marital status, with no significant differences (all *P*-values > 0.05) (Table 1).

**Table 1:** Baseline characteristics of participants.

Demographic and occupational characteristics	High-noise (*n* = 46)	Low-noise (*n* = 48)	*P*
**Age (years), mean ±SD**	30.2 ± 3.6	29.5 ± 4.3	0.635
**BMI (kg/m^2^), mean ±SD**	23.8 ± 4.6	23.1 ± 4.3	0.407
**Gender**			0.730
Male	9 (19.6%)	12 (25.0%)	
Female	37 (80.4%)	36 (75.0%)	
Work experience^*^(years), mean ±SD	6.7 ± 3.9	7.1 ± 4.5	0.765
**Work time per week (hours), mean ±SD**	43.8 ± 6.5	45.3 ± 5.7	0.656
**Education level**			0.630
Diploma	9 (19.6%)	10 (20.8%)	
Bachelor's degree	30 (65.2%)	33 (68.8%)	
Master's degree	7 (15.2%)	5 (10.4%)	
**Marital status**			0.750
Single	16 (34.8%)	12 (25.0%)	
Married	25 (54.3%)	29 (60.4%)	
Divorced/Separated	5 (10.9%)	7 (14.6%)	

BMI, body mass index. ^*^: Work experience refers to experience in the same type of operating room (high-noise or low-noise).

### 3.2 Psychological outcomes

Table 2 summarizes the comparison of self-reported psychological outcomes between groups. Compared with the low-noise group, the high-noise group demonstrated significantly higher scores on anxiety (GAD-7), sleep disturbance (AIS), emotional exhaustion and cynicism (MBI-GS subscales), and perceived stress (PSS-10) (all *P* < 0.001). No significant difference was observed in the professional efficacy subscale of the MBI-GS (*P* = 0.693).

**Table 2:** Comparison of self-reported psychological status between high-noise and low-noise groups.

Psychological measure	High-noise (*n* = 46)	Low-noise (*n* = 48)	Mean difference (95% CI)	Cohen's d (95% CI)	*P*
**GAD-7**	7.64 ± 3.98	4.14 ± 2.35	3.50 (2.17 to 4.83)	1.08 (0.64 to 1.51)	< 0.001
**AIS**	8.50 ± 4.27	5.29 ± 3.45	3.21 (1.62 to 4.80)	0.83 (0.41 to 1.25)	< 0.001
**MBI-GS (exhaustion)**	16.07 ± 7.56	8.00 ± 4.52	8.07 (5.53 to 10.61)	1.30 (0.86 to 1.75)	< 0.001
**MBI-GS (cynicism)**	10.36 ± 7.64	4.43 ± 2.90	5.93 (3.58 to 8.28)	1.03 (0.60 to 1.47)	< 0.001
**MBI-GS (professional efficacy)**	26.30 ± 6.36	28.43 ± 7.88	−2.13 (−5.07 to 0.81)	−0.30 (−0.70 to 0.11)	0.693
**PSS-10**	15.64 ± 5.12	9.71 ± 3.87	5.93 (4.08 to 7.78)	1.31 (0.86 to 1.76)	< 0.001

GAD-7, General Anxiety Disorder-7; AIS, Athens Insomnia Scale; MBI-GS, Maslach Burnout Inventory-General Survey; PSS-10, Perceived Stress Scale; CI, confidence interval.

### 3.3 Physiological parameters and physical symptoms

Table 3 presents the comparison of physiological indicators and self-reported physical symptoms between the high-noise and low-noise groups. In terms of physiological metrics, the high-noise group exhibited significantly higher RHR (*P* = 0.016) and SBP (*P* = 0.039). Although DBP was higher in the high-noise group, the difference did not reach statistical significance (*P* = 0.067). Regarding self-reported physical symptoms, the high-noise group reported a significantly higher prevalence of tinnitus episodes (*P* = 0.013), subjective hearing impairment (*P* = 0.011), and palpitations (*P* = 0.028). However, no significant differences were observed in the rates of gastrointestinal discomfort (*P* = 0.632), headache or dizziness (*P* = 0.114), or musculoskeletal discomfort (*P* = 0.144).

**Table 3:** Comparison of physiological parameters and self-reported physical symptoms between high-noise and low-noise groups.

Parameters and symptoms	High-noise (*n* = 46)	Low-noise (*n* = 48)	Effect size (95% CI)	*P*
**Physiological parameters**			Mean difference (95% CI)/Cohen's *d*	
**RHR, bpm**	80.5 ± 6.9	73.1 ± 8.1	7.40 (4.31 to 10.49); *d* = 0.98 (0.55 to 1.41)	0.016
**SBP, mmHg**	115.3 ± 11.3	106.6 ± 11.2	8.70 (4.09 to 13.31); *d* = 0.77 (0.35 to 1.19)	0.039
**DBP, mmHg**	71.4 ± 6.6	68.9 ± 10.2	2.50 (−1.04 to 6.04); *d* = 0.29 (−0.12 to 0.70)	0.067
**Self-reported Physical Symptoms**			OR (95% CI)	
**Tinnitus episodes**, ***n*** **(%)**	21 (45.6%)	5 (10.4%)	7.22 (2.42 to 21.55)	0.013
**Subjective hearing impairment**, ***n*** **(%)**	30 (65.2%)	12 (25.0%)	5.62 (2.31 to 13.72)	0.011
**Palpitations**, ***n*** **(%)**	12 (26.1%)	3 (6.3%)	5.29 (1.38 to 20.24)	0.028
**Gastrointestinal discomfort**, ***n*** **(%)**	7 (15.2%)	4 (8.3%)	1.97 (0.54 to 7.26)	0.632
**Headache or dizziness**, ***n*** **(%)**	14 (30.4%)	5 (10.4%)	3.76 (1.23 to 11.52)	0.114
**Musculoskeletal discomfort**, ***n*** **(%)**	16 (34.8%)	7 (14.6%)	3.12 (1.14 to 8.54)	0.144

RHR, resting heart rate; bmp, beats per minute; SBP, systolic blood pressure; DBP, diastolic blood pressure. OR, odds ratio; CI, confidence interval. Effect sizes are reported as mean difference with Cohen's d for continuous variables and odds ratios for categorical variables.

### 3.4 Perceived impact on surgical performance

Table 4 presents the subjective perceptions of noise impact in the high-noise and low-noise groups. The high-noise group reported significantly higher perceived impacts on task efficiency (*P* = 0.002), surgical concentration (*P* = 0.019), and intraoperative communication (*P* = 0.032) compared to the low-noise group.

**Table 4:** Comparison of perceived impact of noise on surgical performance between high-noise and low-noise groups.

Surgical performance domain	High-noise (*n* = 46)	Low-noise (*n* = 48)	Mean difference (95% CI)	Cohen's d (95% CI)	*P*
**Task efficiency**	6.71 ± 1.59	4.71 ± 1.44	2.00 (1.38 to 2.62)	1.32 (0.87 to 1.77)	0.002
**Surgical concentration**	6.07 ± 2.13	4.43 ± 1.22	1.64 (0.93 to 2.35)	0.95 (0.52 to 1.38)	0.019
**Intraoperative communication**	6.57 ± 2.03	5.00 ± 1.62	1.57 (0.82 to 2.32)	0.86 (0.43 to 1.28)	0.032

CI, confidence interval.

## 4 Discussion

This study investigated the psychological, physiological, and subjective correlates of working in high-noise vs. low-noise operating room environments among scrub nurses. Psychologically, nurses in the high-noise group reported significantly higher levels of anxiety, sleep disturbance, emotional exhaustion, cynicism, and perceived stress, with effect sizes consistently in the large range. Physiologically, the high-noise group exhibited elevated resting heart rate and systolic blood pressure, accompanied by a markedly higher prevalence of noise-related somatic complaints such as tinnitus, subjective hearing impairment, and palpitations rather than clinically diagnosed conditions. Subjectively, this group also perceived a substantially greater negative impact of noise on task efficiency, surgical concentration, and intraoperative communication. Notably, despite the elevated exhaustion and cynicism, professional efficacy scores were comparable between groups, with the confidence interval crossing zero. This dissociation suggests that while noise exposure imposes a heavy toll on psychological wellbeing, it does not necessarily diminish nurses' perceived professional competence. Taken together, the consistently large effect sizes across multiple domains indicate that the observed differences are not only statistically significant but also clinically meaningful.

Several plausible mechanisms may underlie the observed associations. Chronic intermittent noise exposure is a well-established non-specific stressor that activates the hypothalamic-pituitary-adrenal axis and the sympathetic-adrenomedullary system. The resulting sustained elevations in cortisol and catecholamines can manifest as elevated resting heart rate, increased systolic blood pressure, anxiety, and sleep disturbance, a pattern consistent with our findings (13, 19). Cognitively, peak operative noise imposes substantial extraneous load on auditory working memory and selective attention, which may explain the perceived deterioration in task efficiency, surgical concentration, and intraoperative communication in the high-noise group. This aligns with experimental evidence that intraoperative noise impairs auditory processing and laparoscopic performance in surgeons (11, 16, 17). The dissociation between elevated exhaustion and cynicism but preserved professional efficacy is also theoretically coherent. According to the conservation-of-resources and Maslach burnout frameworks, the energetic and attitudinal components of burnout typically deteriorate before the self-evaluative component, particularly in highly trained professionals whose technical identity remains robust. Repeated tinnitus and subjective hearing impairment, in turn, likely reflect temporary threshold shifts and auditory fatigue, which are established precursors of cumulative noise-induced hearing loss (10, 18–20).

Our findings extend prior work in several important ways. Although studies on surgeons (10, 11, 16, 17, 19), anesthesiologists (5, 18, 20), and patients (21–24) have highlighted the adverse effects of operating room noise, evidence specific to scrub nurses remains scarce. Keller et al. (14) reported that scrub nurses experienced the highest distraction rate (96.4%) among operating room personnel during 110 abdominal surgeries, and Padmakumar et al. (15) found in a UK-wide survey of 519 healthcare professionals (including 37 nurses) that the majority believed noise contributed to errors and impaired communication and concentration. Gülşen et al. (19) further documented physiological and psychological effects of ambient noise across mixed surgical staff. However, these studies were uncontrolled cross-sectional surveys that did not isolate scrub nurses or compare contrasting noise environments, potentially failing to capture the true occupational burden borne by this group. To the best of our knowledge, this is the first study to directly compare scrub nurses working in well-defined high- and low-noise environments while triangulating psychological, physiological, and perceived-performance domains. Our findings are broadly consistent with previous reports on surgeons and anesthesiologists (2, 6, 34) and provide more targeted evidence that scrub nurses bear a substantive and previously underappreciated occupational burden. These results also underscore the need for further research on whether noise-reduction strategies, shown to benefit other healthcare professionals (35), can yield comparable benefits for scrub nurses.

These findings carry several actionable implications. First, noise-intensive specialties such as orthopedics and otolaryngology should integrate routine noise monitoring into operating room quality assurance, supported by engineering controls (e.g., low-noise instruments, acoustic dampening) and administrative measures such as “sterile cockpit” periods during critical procedural phases. Second, scrub nurses chronically assigned to high-noise rooms warrant inclusion in formal hearing conservation programs with baseline and periodic pure-tone audiometry. Third, occupational health services should incorporate routine screening for anxiety, sleep disturbance, and burnout in this subgroup, with timely referral pathways. Finally, if longitudinal evidence confirms cumulative health risk, recognizing high-noise operating rooms as occupationally hazardous environments would be a logical institutional response. Corresponding measures may include hazard allowances, mandatory rotation policies, or staffing models that limit cumulative exposure, in line with occupational health principles already applied to other hospital noise-exposed populations.

This study has several limitations. First, the cross-sectional design limits the ability to draw causal conclusions about the relationship between noise exposure and health outcomes, necessitating longitudinal studies to further explore the cumulative, long-term effects of high-noise environments. Second, a notable limitation is the predominant reliance on self-reported measures across psychological, physiological, and performance domains, which introduces a degree of subjectivity and lacks objective clinical validation. Specifically, regarding psychological and performance outcomes, although validated scales were used, the results reflect participants' self-perception rather than clinical psychiatric diagnoses, and the reported adverse impacts on surgical performance represent perceived impairments in concentration and communication rather than objective operational metrics such as error rates or procedure duration. Similarly, the assessment of physical health lacked clinical corroboration. For instance, hearing difficulties were evaluated based on subjective perception rather than standard pure-tone audiometry, making it impossible to distinguish between temporary auditory fatigue and permanent noise-induced hearing loss, nor were palpitations verified by real-time electrocardiogram monitoring. Third, despite pre-specified reporting criteria (recurrent symptoms ≥2–3 times/week during or within 2 h after surgical shifts), the symptom assessment relied on 12-month retrospective self-report without corroboration from symptom diaries, medical records, or clinical examinations. Selective recall or over-attribution of symptoms to occupational noise in the high-noise group therefore cannot be excluded. Prospective designs using real-time symptom diaries and objective clinical verification are warranted in future research. Fourth, complete researcher blinding was not achievable given the obvious environmental differences between the two settings. Although standardized self-administered questionnaires and automated measurement devices were used to mitigate observer bias, residual assessment bias remains possible. Finally, the sample size of per group remains relatively small, which may limit the statistical power and the generalizability of the findings to broader surgical settings. Therefore, larger, multicenter studies with objective physiological monitoring are recommended for future research.

## 5 Conclusion

The findings suggest that prolonged exposure to high-noise environments is associated with elevated psychological distress, physiological stress responses, and frequent somatic complaints among scrub nurses. Additionally, high noise levels are perceived to negatively interfere with surgical performance. Longitudinal studies and targeted noise-reduction interventions are recommended to safeguard occupational health and patient safety.

## Data Availability

The raw data supporting the conclusions of this article will be made available by the authors, without undue reservation.

## References

[1] Ayoola M Abelleyra Lastoria DA Casey L Dardak S Rupra R Hing CB . Noise in operating theatres, is it safe? Arch Orthop Trauma Surg. (2024) 144:3343–9. doi: 10.1007/s00402-024-05489-x 39105841 PMC11417073

[2] Katz JD. Noise in the operating room. Anesthesiology. (2014) 121:894–8. doi: 10.1097/ALN.0000000000000319 24878496

[3] Siverdeen Z Ali A Lakdawala AS McKay C. Exposure to noise in orthopaedic theatres–do we need protection? Int J Clin Pract. (2008) 62:1720–2. doi: 10.1111/j.1368-5031.2007.01689.x 19143857

[4] Sydney SE Lepp AJ Whitehouse SL Crawford RW. Noise exposure due to orthopedic saws in simulated total knee arthroplasty surgery. J Arthroplasty. (2007) 22:1193–7. doi: 10.1016/j.arth.2007.05.048 18078890

[5] Wahr JA Abernathy JH 3rd. Too loud to hear myself think: deleterious effects of noise in the operating room. Br J Anaesth. (2024) 132:840–2. doi: 10.1016/j.bja.2024.02.007 38448271

[6] Louis M Grabill N Strom P Gibson B. Leading through noise: operating room noise challenges for staff and leadership techniques to ensure optimal operational performance. Cureus. (2024) 16:e69569. doi: 10.7759/cureus.69569 39421089 PMC11484183

[7] Arabaci A Önler E. The effect of noise levels in the operating room on the stress levels and workload of the operating room team. J Perianesth Nurs. (2021) 36:54–8. doi: 10.1016/j.jopan.2020.06.024 33077358

[8] Kracht JM Busch-Vishniac IJ West JE. Noise in the operating rooms of Johns Hopkins hospital. J Acoust Soc Am. (2007) 121:2673–80. doi: 10.1121/1.2714921 17550167

[9] Sampieri G Namavarian A Levin M Philteos J Lee JW Koskinen A . Noise in otolaryngology - head and neck surgery operating rooms: a systematic review. J Otolaryngol. (2021) 50:8. doi: 10.1186/s40463-020-00487-6 PMC7879658 33573705

[10] McLeod R Myint-Wilks L Davies SE Elhassan HA. The impact of noise in the operating theatre: a review of the evidence. Ann R Coll Surg Engl. (2021) 103:83–7. doi: 10.1308/rcsann.2020.7001 33559553 PMC9773860

[11] Way TJ Long A Weihing J Ritchie R Jones R Bush M . Effect of noise on auditory processing in the operating room. J Am Coll Surg. (2013) 216:933–8. doi: 10.1016/j.jamcollsurg.2012.12.048 23518255

[12] Grenzebach J Romanus E. Quantifying the effect of noise on cognitive processes: a review of psychophysiological correlates of workload. Noise Health. (2022) 24:199–214. doi: 10.4103/nah.nah_34_22 36537445 PMC10088430

[13] Basner M Babisch W Davis A Brink M Clark C Janssen S . Auditory and non-auditory effects of noise on health. Lancet. (2014) 383:1325–32. doi: 10.1016/S0140-6736(13)61613-X 24183105 PMC3988259

[14] Keller S Tschan F Beldi G Kurmann A Candinas D Semmer NK. Noise peaks influence communication in the operating room. An observational study. Ergonomics. (2016) 59:1541–52. doi: 10.1080/00140139.2016.1159736 27054273

[15] Padmakumar AD Cohen O Churton A Groves JB Mitchell DA Brennan PA. Effect of noise on tasks in operating theatres: a survey of the perceptions of healthcare staff. Br J Oral Maxillofac Surg. (2017) 55:164–7. doi: 10.1016/j.bjoms.2016.10.011 27810115

[16] Moorthy K Munz Y Dosis A Bann S Darzi A. The effect of stress-inducing conditions on the performance of a laparoscopic task. Surg Endosc. (2003) 17:1481–4. doi: 10.1007/s00464-002-9224-9 12820061

[17] Moorthy K Munz Y Undre S Darzi A. Objective evaluation of the effect of noise on the performance of a complex laparoscopic task. Surgery. (2004) 136:25–30. doi: 10.1016/j.surg.2003.12.011 15232535

[18] Crockett CJ Dalal PG Tjia I Allen M Edelstein E Feng X . Loud call for silence: anaesthesiologists' perceptions of noise in the operating room. Br J Anaesth. (2024) 132:444–7. doi: 10.1016/j.bja.2023.11.035 38101964

[19] Gülşen M Aydingülü N Arslan S. Physiological and psychological effects of ambient noise in operating room on medical staff. ANZ J Surg. (2021) 91:847–53. doi: 10.1111/ans.16582 33459517

[20] Yu CV Foglia J Yen P Montemurro T Schwarz SKW MacDonell SY. Noise in the operating room during induction of anesthesia: impact of a quality improvement initiative. Can J Anaesth. (2022) 69:494–503. doi: 10.1007/s12630-021-02187-9 35014000

[21] Hasfeldt D Maindal HT Toft P Birkelund R. Patients' perception of noise in the operating room–a descriptive and analytic cross-sectional study. J Perianesth Nurs. (2014) 29:410–7. doi: 10.1016/j.jopan.2014.03.001 25261144

[22] Peisl S Sánchez-Taltavull D Guillen-Ramirez H Tschan F Semmer NK Hübner M . Noise in the operating room coincides with surgical difficulty. BJS open. (2024) 3:8. doi: 10.1093/bjsopen/zrae098 PMC11482277 39413049

[23] Dholakia S Jeans JP Khalid U Dholakia S D'Souza C Nemeth K. The association of noise and surgical-site infection in day-case hernia repairs. Surgery. (2015) 157:1153–6. doi: 10.1016/j.surg.2014.12.026 25737006

[24] Kurmann A Peter M Tschan F Mühlemann K Candinas D Beldi G. Adverse effect of noise in the operating theatre on surgical-site infection. Br J Surg. (2011) 98:1021–5. doi: 10.1002/bjs.7496 21618484

[25] Löwe B Decker O Müller S Brähler E Schellberg D Herzog W . Validation and standardization of the generalized anxiety disorder screener (GAD-7) in the general population. Med Care. (2008) 46:266–74. doi: 10.1097/MLR.0b013e318160d093 18388841

[26] Spitzer RL Kroenke K Williams JB Löwe B. A brief measure for assessing generalized anxiety disorder: the GAD-7. Arch Intern Med. (2006) 166:1092–7. doi: 10.1001/archinte.166.10.1092 16717171

[27] Fabbri M Beracci A Martoni M Meneo D Tonetti L Natale V. Measuring subjective sleep quality: a review. Int J Environ Res Public Health. (2021) 26:18. doi: 10.3390/ijerph18031082 PMC7908437 33530453

[28] Dall'Ora C Ball J Reinius M Griffiths P. Burnout in nursing: a theoretical review. Hum Res Health. (2020) 18:41. doi: 10.1186/s12960-020-00469-9 PMC7273381 32503559

[29] Cohen S Kamarck T Mermelstein R. A global measure of perceived stress. J Health Soc Behav. (1983) 24:385–96. doi: 10.2307/2136404 6668417

[30] Bai M Jing T Li S Zhang Z. Unravelling critical burnout syndrome in Chinese hospitals a network analysis of the Maslach burnout inventory human services survey. Sci Rep. (2025) 15:9870. doi: 10.1038/s41598-025-93627-9 40118901 PMC11928527

[31] Chung KF Kan KK Yeung WF. Assessing insomnia in adolescents: comparison of insomnia severity index, athens insomnia scale and sleep quality index. Sleep Med. (2011) 12:463–70. doi: 10.1016/j.sleep.2010.09.019 21493134

[32] Jiang C Ma H Luo Y Fong DYT Umucu E Zheng H . Validation of the Chinese version of the perceived stress scale-10 integrating exploratory graph analysis and confirmatory factor analysis. Gen Hosp Psychiatry. (2023) 84:194–202. doi: 10.1016/j.genhosppsych.2023.07.008 37572467

[33] Tong X An D McGonigal A Park SP Zhou D. Validation of the generalized anxiety disorder-7 (GAD-7) among Chinese people with epilepsy. Epilepsy Res. (2016) 120:31–6. doi: 10.1016/j.eplepsyres.2015.11.019 26709880

[34] Hasfeldt D Laerkner E Birkelund R. Noise in the operating room–what do we know? A review of the literature. J Perianesth Nurs. (2010) 25:380–6. doi: 10.1016/j.jopan.2010.10.001 21126668

[35] Engelmann CR Neis JP Kirschbaum C Grote G Ure BM. A noise-reduction program in a pediatric operation theatre is associated with surgeon's benefits and a reduced rate of complications: a prospective controlled clinical trial. Ann Surg. (2014) 259:1025–33. doi: 10.1097/SLA.0000000000000253 24394594

